# Heat-Related Hospitalizations in Older Adults: An Amplified Effect of the First Seasonal Heatwave

**DOI:** 10.1038/srep39581

**Published:** 2017-01-11

**Authors:** Alexander Liss, Ruiruo Wu, Kenneth Kwan Ho Chui, Elena N. Naumova

**Affiliations:** 1 Department of Civil and Environmental Engineering, Tufts University School of Engineering, Medford, MA 02155, USA; 2 Initiative for the Forecasting and Modeling of Infectious Diseases, Tufts University, Medford, MA 02155, USA; 3 Department of Public Health and Community Medicine, Tufts University School of Medicine, Boston, MA 02111, USA; 4 Friedman School of Nutrition Science and Policy, Tufts University, Boston, MA 02111, USA

## Abstract

Older adults are highly vulnerable to the detriment of extreme weather. The rapid non-linear increase in heat-related morbidity is difficult to quantify, hindering the attribution of direct effects of exposure on severe health outcomes. We examine the effects of ambient temperatures on heat-related hospitalizations (HH) among the elderly in presence of strong seasonality and by assessing the effects caused by the first and subsequent seasonal heatwaves. We empirically derived the thresholds for a heatwave episode in Boston MSA based on 16 years of daily observations. We compared the health risks of heatwaves using the proposed and four alternative definitions. 701 cases of HH in older residents of Boston area were examined using harmonic regression models, designed to capture the non-linear effects of ambient temperatures and heatwave episodes when the night-time temperature is above 65.5 °F for 3 consecutive nights. The overall relative risk of HH associated with a heatwave episode was 6.9 [95%CI:4.8–9.8]. The relative risk of HH associated with the first heatwave increases up to 13.3 [95%CI:7.4–24.0]. The risk declined to 3.7 [95%CI:2.4–5.8] for the subsequent heatwave. Four other commonly used heatwave definitions confirmed these findings. Public health actions have to target the first heatwave to maximize the impact of preventive measures.

Heat waves threaten health, especially of those who are less capable to cope and adapt to the thermal extremes^1^,^2^. The recent WHO report and the 2016 US Global Change Research Program on Human Health emphasized the importance of this issue^3^,^4^. The major heat wave in Europe in the summer of 2003 caused 15,000 deaths in France alone^5^,^6^,^7^,^8^,^9^ and has prompted several investigations of the relationship between human health and maximum daily ambient temperature^10^,^11^,^12^. It has been reported that elderly, defined as people who are 65 years or older, suffer disproportionally more than younger population during heat waves due to lower efficiency of their thermoregulatory mechanisms, potential side effects of medications, and limited mobility^13^,^14^,^15^,^16^,^17^,^18^. Furthermore, high perspiration threshold combined with increased blood viscosity, elevated cholesterol level, and diminished ability to detect changes in body temperature may further contribute to severe heat-related health conditions in older adults. The older adult population in the USA is increasing in both size and proportion^19^, underpinning the need of targeted preventive actions^20^.

While the body of research on adverse health effects of extreme weather is rapidly growing, some methodological issues of assessing such effects have yet to be addressed. One of the major ongoing issues is the definition of a heatwave. The WHO report^3^ lists at least fifteen different exposure metrics used to categorize heat exposure. The Report on the Impacts of Climate Change on Human Health in the United States also notes the lack of commonly accepted methodology in defining extreme hot weather: “*Extremes can be defined by average, minimum, or maximum daily temperatures, by nighttime temperatures, or by daytime temperatures. However, there is no standard method for defining a heat wave or cold wave. There are dramatic differences in the observed relationships between temperature, death, and illness across different regions and seasons; these relationships vary based on average temperatures in those locations and the timing of the heat or cold event*”^4^. Recent studies provide extensive and detailed overviews and comparisons of various indicators of extreme heat events^21^,^22^,^23^,^24^. It is apparent that the most promising and realistic measures consider both duration and intensity of exposure^21^,^22^,^23^,^24^, so the heatwave indicators mark when the value of a specific measure exceeds an absolute or relative threshold for a selected environmental condition lasting for some period of time, usually from 1 to 5 days. Examples of such definitions include situations when a heat wave is defined as “environmental condition when the daily maximum temperature of more than five consecutive days exceeds the average maximum temperature by 5 °C (9 °F)”^25^, or “when minimum daily temperature exceeds 95^th^ percentile for 2 consecutive nights”^21^.

The heatwave threshold is typically determined using absolute values of used measures (physiology-based threshold), as well as relative values (location-based threshold), such as 81^st^ to 99^th^ percentiles^26^. Physiology-based thresholds are linked to comfort-related ergonomic conditions with potentially less narrow range than location-based thresholds, which vary dramatically and thus reduce comparability of research findings. Yet, the concordance between physiology-based and location-based thresholds is rarely established.

The decision for selecting physiology-based or location-based thresholds in specific context is not clear, yet a solution can be found in better understanding the non-linear relationship between ambient temperature and health conditions. Heat-related morbidity and temperature typically shows a J-shaped relationship with shapes varying by location, climate features, and affected populations^16^,^27^,^28^,^29^,^30^,^31^,^32^. The lower part of the “J” shape indicate a thermal “comfort” zone, in which heat-related morbidity are less likely to occur. Above the comfort zone, the associated increase in mortality with a unit of exposure increase accelerates. A better characterization of such non-linear relationships should advance the detection of meaningful thresholds and the formulation of location-specific physiologically relevant definition of heatwave episodes.

Ability to quantify and differentiate the effect of individual heat waves with respect to their time of appearance represents another important issue. If the early heat waves pose elevated threat to public, more emphasis could be made on protective measures at the onset of warm season^33^,^34^,^35^. A disproportional effect of the early season’s hot weather on mortality has been noted^33^,^34^. A study in North Italian province of Veneto has demonstrated that morbidity equally peaked at the first and the last heat waves of the season^35^. Thus, if such a phenomenon holds uniformly especially in vulnerable population, effective communication, and mitigation strategies can be better tailored.

While the heat-related mortality has been widely discussed, less attention has been paid to morbidity due to the limited access to reliable data, complexity of reporting, and multifaceted response to the heat. The benefits of using Medicare claim data for large scale investigations of the vulnerable older populations due to its universal, near-exhaustive coverage of Medicare beneficiaries aged 65 and above are well demonstrated^36^,^37^. Using this large national data repository comprising of approximately 220 million individual records, we examined the effects of maximum daily ambient temperature on hospitalizations caused by heat exposure among adults residing in the Boston Metropolitan Statistical Area (MSA) between January 1^st^ 1991 and December 31^st^ 2006, inclusive. These urban communities of Massachusetts are characterized by temperate climate, relatively high living standards, close proximity and easy access to points of medical care. We hypothesized that: (1) the magnitude and duration of ambient temperature exposure directly contributes to the occurrence and severity of HHs in a non-linear fashion with an accelerating effect when ambient day and night temperatures exceeded specific thresholds; (2) the first heatwave of each year is associated with more cases of HHs when compared to the subsequent heatwaves in the same year; and (3) the disproportional effect of the first wave will be present after adjusting for the seasonal nature of exposure the HHs has well-pronounced temporal features, which can be described by harmonic oscillations and specific calendar effects. We empirically defined location-specific thresholds and describe non-linear associations between daily ambient temperature and hospitalization rates due to exposure to environmental heat based on International Classification of Diseases (ICD-9-CM). We determined the effect caused by the first and subsequent heatwaves by estimating the relative risks in presence of well-pronounced seasonal variations of the selected health outcomes. We then compared the proposed data-driven definition with four commonly used definitions of a heatwave episode in terms of assessing the detrimental effects on heat-related hospitalizations.

## Methods

### Hospitalization records

**Figure 1:**
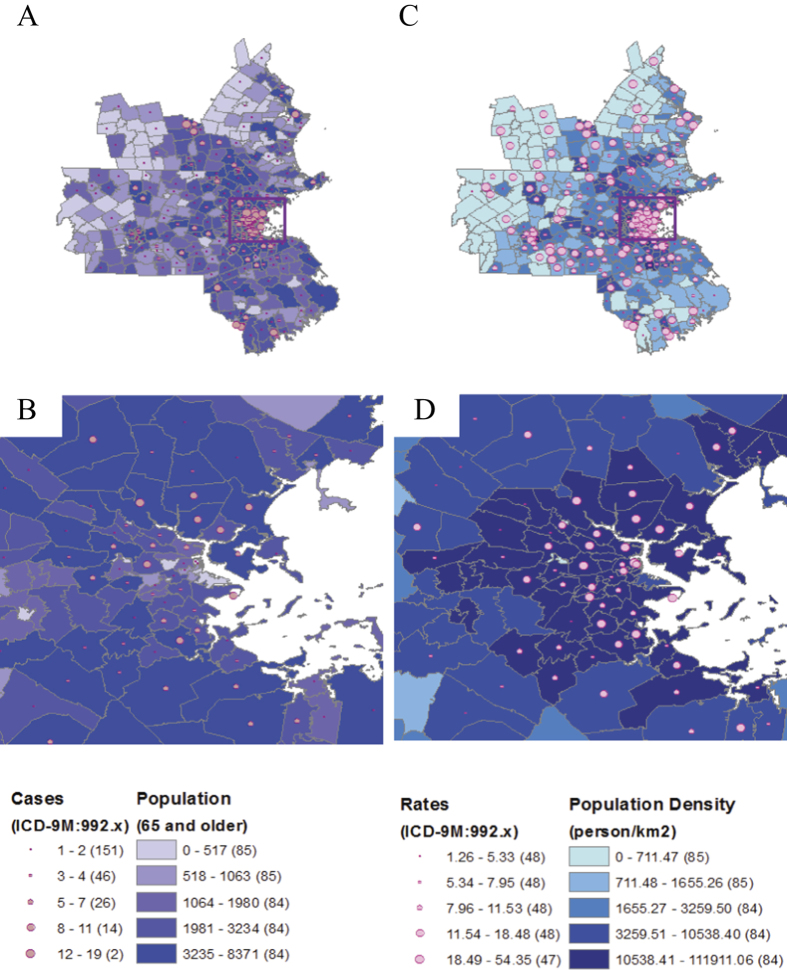
The distributions of heat-related hospitalizations (HH), population of older adults aged 65 and over, or elderly (Panels (**A**) and (**B**), respectively), hospitalization rates (per 1 M people) and elderly population density (Panels (**C**) and (**D**), respectively), within the Boston MSA (Panels (**A**) and (**C**), respectively), and its the most urbanized part (Panels (**B**) and (**D**), respectively) observed in 1991–2006. Maps were created with ArcGIS 10.2. http://www.esri.com/software/arcgis/arcgis-for-desktop.

Daily hospitalization records from January 1^st^ 1991 to December 31^st^ 2006 were obtained from the CMS database. Each record contains age, ZIP code of residence, date of admission, and up to 10 diagnostic codes based on International Classification of Disease, 9^th^ Revision, Clinical Modification (ICD-9-CM). 1123 hospitalization records with ICD-9-CM 992.0–992.9 in any of 10 diagnostic fields were abstracted. This ICD category covers a broad range of health conditions, which in the opinion of a treating physician are most likely caused by environmental exposures to heat. While other ICD categories are associated with exposure to hot weather, we purposefully selected this ICD codes to minimize potential misclassification. For 701 records selected for the analysis, the heat-related diagnosis was listed as primary or secondary cause; 83% of cases were coded as heat stroke or heat exhaustion (Table 1). Using reported ZIP codes of residence we selected all ZIP codes that belongs to Boston-Cambridge-Quincy Metropolitan Statistical Area (Boston MSA) (United States Census Bureau 2014). According to the US Office of Management and Budget, MSA is defined as “a region that has at least one urbanized area of 5,000 or more population, plus adjacent territory that has a high degree of social and economic integration with the core as measured by community ties.” In 2010, Boston MSA was the 10^th^ most populated MSA in the US with the total area of 4,674 mi^2^ and the total population of 4,552,402; consisted of 74.9% non-Hispanic White population, 9% Hispanic and Latino population, 7.4% non-Hispanic Black population, 7.1% Asians and 1.6% of other races and ethnicities; 10% of MSA population was 65 years old and older (United States Census Bureau 2014). Using linear interpolation of Census 1990, 2000, and 2010 data we estimated older adult population in the study area, calculated the annual hospitalization rates per 1 million people aged 65 and older. We created the MSA maps by matching ZIP codes of the Census basemaps with the list of ZIP codes within Boston MSA and mapped the hospitalizations with ArcGIS 10.2. http://www.esri.com/software/arcgis/arcgis-for-desktop (see Fig. 1).

### Ambient temperature data

Daily temperature records of the Boston MSA were obtained from the National Oceanic and Atmospheric Administration–Global Summary of the Day (NOAA-GSOD) (Unites States National Oceanic and Atmospheric Administration 2014) for the study period. The dataset includes maximum and minimum daily temperature from 83 meteorological stations situated within the borders of Boston MSA and up to 120 miles buffer zone. The daily temperature data were interpolated for each ZIP code (average number of stations per ZIP code: 54.4 ± 4.7, distance between nearest station and ZIP code centroid: 7.75 ± 3.84 miles) using an inverse distance weighting (IDW) method, which allows for multivariate interpolation by assigning the values to unknown locations calculated with a weighted average of the values available at the known points^38^.

### Non-linear fit and empirical definition of a heatwave episode

To capture the effect of a heatwave on non-linear exponential increase in hospitalization counts we derived an empirical definition of a heatwave episode assuming that the magnitude and duration of ambient temperature exposure directly contributes to the occurrence and severity of heat-related hospitalizations (HHs) in a non-linear fashion with an accelerating effect when ambient day and night temperatures exceeded specific thresholds.

To estimate thresholds, the maximum (day-time) and minimum (night-time) temperature values were transformed and parameterized as follow:


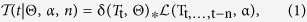


where δ(T_t_, Θ) is a Dirac delta function with a threshold parameter Θ, and 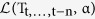 is a scaled lag-distributed ambient temperature for *n* periods with exponential decay parameter *α*.

Dirac delta function *δ*(T_t_, Θ) in Eq. (1) is defined as:


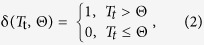


where T_t_ is the daily temperature value for a t-day and Θis a temperature threshold value.

Scaled lag-distributed temperature 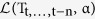 in Eq. (1) is defined as weighted sum of temperature measures for *n* days prior to date *t*, linearly scaled to the [0..1] interval:


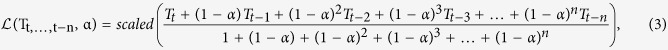


where *α* is the exponential decay parameter and *n* is the number of included temporal lags. Due to the exponential nature of weights *α* in Eq. (3), the effective number of lags *n*, i.e. the number of days with sufficiently large weight capable to substantially influence the outcome, is determined as:


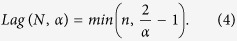


By setting parameter *n* sufficiently large, the exponential decay parameter *α* effectively determines the number of lags included in the model. For example, with the number of lags *n* = 10, and decay parameter *α* = 0.5, the contribution of lag 0 is slightly above 50%, the contribution of lag 4 is about 6%, and the contribution of lag 10 is less than 0.1%.

To fit the non-linear association between daily ambient temperature and HH the Negative-Binomial Generalized Linear Model (NB-GLM) has been applied:


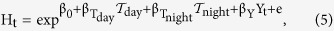


where H_t_ is the daily hospitalization counts for the study period; 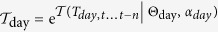, 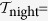 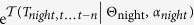 and *Y*_*t*_ is an indicator variable absorbing inter-year variability. We selected the vector of parameters:


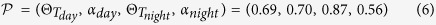


by maximizing goodness of fit of the NB-GLM Eq. (5)) based on Akaike Information Criterion (AIC). The optimized data-driven parameters Θ and *α* from Eq. (6) represent temperature threshold and effective duration or a number of days with temperature above this threshold that maximizes the non-linear effect of the scaled lag-distributed ambient temperature on the health outcome. As such, the parameter vector 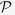
can be used to determine heatwave episodes. The length of the effective day time lag is 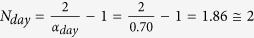, and the effective night-time temperature lag is 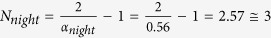. Thus, we proposed a definition of heatwave for our study area as an environmental condition when daytime temperature is above 69.5 °F (20.8 °C) for the current and previous day and night-time temperature is above 65.5 °F (18.6 °C) for the current and two previous nights. The span between two consecutive heatwave episodes should be no less than 2 days. A binary variable indicating a day when such condition is met, or a heatwave day, was created for each day during the study period.

### Alternative definitions of a heatwave episode

In addition to the definition of a heatwave proposed above we have examined four alternative heatwave definitions:

Definition A: daily maximum temperature over 95% threshold for 3+ consecutive days^3^;

Definition B: daily maximum temperature over 95% threshold (computed over summer months between May 1 and September 30) for 3+ consecutive days^21^,^26^;

Definition C: maximum daily temperature above 80.6 °F (27 °C) for 6+ consecutive days^3^,^39^,^40^;

Definition D: humidex above 104 °F (40 °C) for 2+ consecutive days^41^,^42^.

Definitions A–C are based on the simple threshold of one parameter (maximum daily ambient temperature), while Definition D is a function of maximum daily temperature and humidity. The first two definitions use relative measures, while the last two use absolute thresholds. We then created binary variables indicating day when relevant conditions are met for each day during the study period to further use in the analysis.

### Estimation of seasonal peaks for heat-related hospitalizations and ambient temperature

The heat related hospitalizations are highly seasonal phenomenon where of the 701 cases of heat-related admissions, 621 (89%) occurred in summer. The harmonic regression has the ability to naturally adjust for periodic seasonal oscillations by using data for the entire study period. A harmonic component properly accounts for transitional periods of spring and autumn and accommodates periods with high level of outcomes (i.e. during the hot season) and with low level of outcome (i.e. during the cold season)^43^,^44^.

First, we fit a NB-GLM to HH counts, denoted as Model 1:


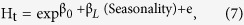


where H_t_ is the daily HH counts for a t-day; β_L_ is the vector of coefficients for a seasonal pattern based on one harmonic term with the period ω = 1/365.25 and Seasonality is a short hand for β_s_sin(2πωt) + β_c_cos(2πωt).

Similarly, daily maximum and minimum temperature values were fitted as Gaussian OLS, as Model 1 modification of:


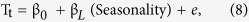


where T_t_ is the daily maximum or minimum temperature value for a t-day; β_L_ is the vector of coefficients for a seasonal pattern based on one harmonic term with the period ω = 1/365.25 and Seasonality ~β_*s*_sin(2πωt) + β_*c*_cos(2πωt).

Based on models’ β_L_ values related to seasonal harmonics, the average peak timing of HH and its 95^th^ confidence interval (CI) were estimated using δ–method^43^,^44^. Peak timing of a periodic process of the form 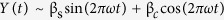 was determined as:


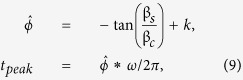


where constant *k* depends on the join sign of estimated coefficients β_*s*_ and β_*c*_: *k* = 0 when both β_*s*_ and β_*c*_ are positive, *k* = 2*π* when β_*s*_ > 0 and β_*c*_ < 0, and *k* = *π* otherwise.

The 95^th^ confidence interval (CI) of the peak timing was determined as:


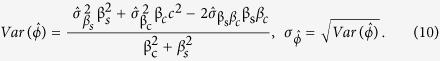


From the Model 1 we determined the peak timing of HH and compared it with the peak timing of the ambient temperature.

### Individual effects of consecutive heat waves

In order to test the stated hypotheses we built statistical models sequentially. First, we built a model considering temporal features in HH time series, such as seasonality, annual and weekly cycles, and the effects of local social calendars (Model 2). Then, we incorporated the heatwave indicator based on the proposed and alternative definitions to test if heatwave days have higher HH than non-heatwave days (Model 3). Finally, we used separate indicators for the first heatwave episode and consecutive episodes during the same season (Model 4). This model allowed us to test the hypothesis that first heatwave have disproportionally large effect on vulnerable population relative to the consecutive heatwaves the same season. These steps are described in details below.

Temporal features in the daily time series of HH counts were examined using NB-GLM for count data, denoted as Model 2:


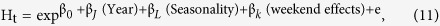


where H_t_ is the daily HH counts; β_J_ is the vector of coefficients for the effect of a calendar year based on a set of indicator variables with 1991 set as a reference year (the β_J_ term accounts for the inter-year changes in population at risk, as well as potential changes in reporting policies and practices during the 16 year study); β_L_ is the vector of coefficients for a seasonal pattern based on a harmonic regression with the period ω = 1/365.25 and two harmonics:


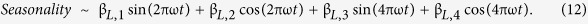


Model 2 also includes variables to account for the effects of weekdays: β_k_ is the vector of coefficients for an indicator variable considering weekends combined with major federal holidays (Saturday, Sunday and major holidays, including New Year’s Day (January 1^st^), Labor Day (June 19^th^), Independence Day (July 4^th^), Veterans Day (Nov 11^th^), and Christmas Day (December 25^th^); weekdays, Monday to Friday, were set as a reference category).

To assess the effect of heatwave episodes we added an indicator variable to Model 2, as in Model 3:


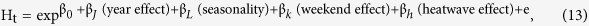


where β_h_ is the coefficient indicating the effects of the heat waves in the season relative to the rest of the days; the remaining coefficients are the same as in Eq. (11).

We then further separated the effect of the first heatwave in the season from the effects of the following up heat waves on HH in Model 4:


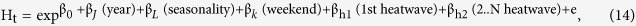


where β_h1_ is the coefficient indicating the effects of the first heatwave in the season relative to the rest of the days, and β_h2_ is the coefficient indicating the effects of the second and all subsequent heat waves in the season relative to the all other days. The relative risk of HH associated with a heatwave episode along with its 95^th^ CI were estimated as: 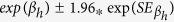 for *β*_*h*1_ and *β*_*h*2_, respectively. Model 4 was also applied to estimate individual effects of the first and consecutive heatwaves on HH using four alternative definitions as described above.

We examined the individual contribution of the effects of the year, seasonality, effects of weekdays and the effects of heatwaves on variability explained by Models 2, 3 and 4 based on the relationship between total and residual deviance and the AIC score.

## Results

**Figure 2:**
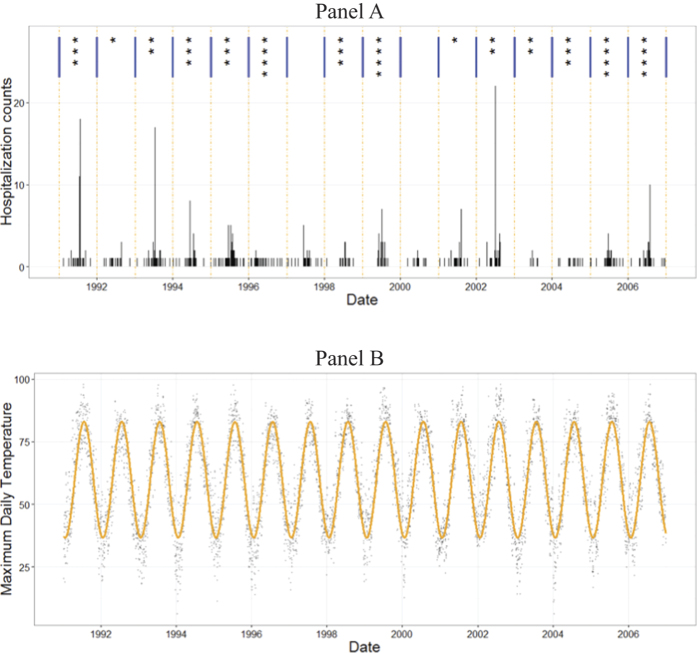
Annual number of heat waves episodes (top row, Panel **A**) and daily counts of heat-related hospitalizations (bottom row, Panel **B**) and daily maximum temperature: actual and fitted with Model B (°F; Panel C) in Boston MSA, 1991–2006.

During the 16-year study period there were 701 hospitalizations due to heat in the Boston-Cambridge-Quincy, MA-NH MSA. Figure 1 illustrates the spatial patterns of the abstracted records. Elevated rates of heat-related hospitalization tend to concentrate in urban areas with greater population density. The time series of daily counts of HH reflects sharp spikes with up to 20-fold increase during summer months (Fig. 2). As estimated from Model 1, HH peaked on July 13^th^ with a median peak date at 194^th^ (IQR: 170^th^; 210^th^) Julian calendar day. As estimated from regression model, ambient maximum and minimum temperature peaked on July 24^th^ (205^th^; IQR: 183^rd^; 213^th^) and on August 1^st^ with a median of 213^th^ (IQR: 205^th^; 243^rd^) Julian calendar day, respectively. Therefore, heat-related hospitalizations are expected to peak on average 11 days earlier than the expected peak in temperature.

**Figure 3:**
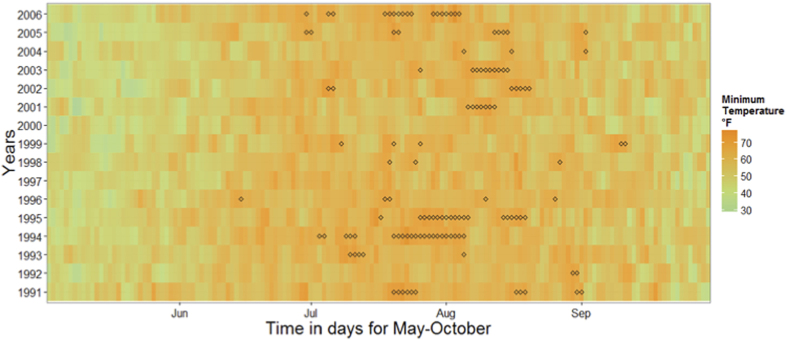
Calendar map of night temperature with “◊” marking days of a heatwave in Boston MSA, 1991–2006 (May through October) based on the proposed definition.

**Figure 4:**
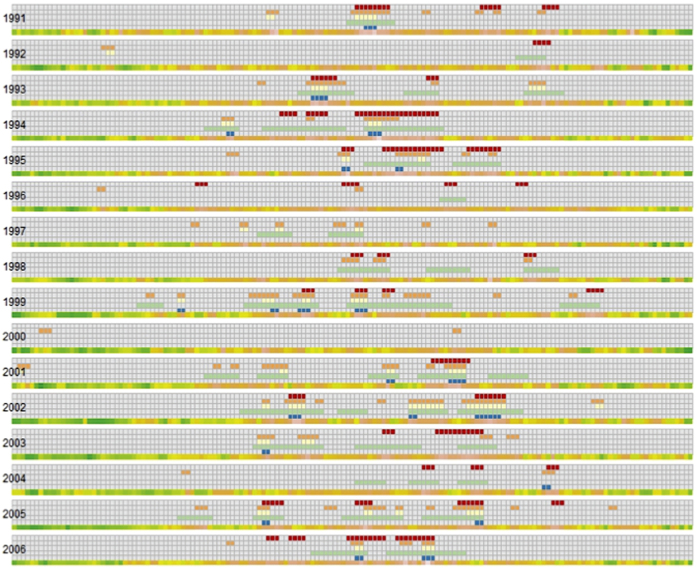
Comparison of the proposed definition with four alternative definitions. Each row represents days from May 1^st^ to Sep 30^th^; Days assigned to be a HW by a selected definition are marked by color (Proposed Definition–red; Definition A–orange; Definition B–yellow, Definition C–green; Definition D–blue). The last line represents maximum daily temperature for a given day for each year with the spectrum from green (~30 °F) to orange (~90 °F).

Based on the proposed definition of heatwave in Boston MSA, we identified 44 heat waves comprised of totally 111 days during the study period (Table 2). Timing for the individual heatwave days along with the heat map of daily night temperature are presented in Fig. 3. Descriptive statistics of heatwave occurrence, duration, the start of the first and last episodes and heat-related hospitalizations (HH) during the heatwave episodes are shown in Table 3. The number of heatwaves and days associated with heatwaves varied for alternative definitions, yet they uniformly agreed on low counts of HH for relatively cold summers of 1992, 1996, 2000, and 2004 (Fig. 4). As expected, Definitions A and D appeared to be the most and least conservative estimates for Boston MSA, respectively.

Over the study period, there were 111 days marked as heatwave days based on the proposed data-driven definition. While they represent only 1.9% out of 5844 days of observations, during those days 207 cases of heat-related hospitalizations were recorded, accounting for 30% of all 701 cases or 33% of 621 cases occurred during the summer-time period. During the first heatwave of the season, that occurred on average on 196.7 ± 17.6 Julian calendar day, almost two weeks before the average peak in minimum temperature, the daily number of hospitalizations were almost 5 times higher as compared to rates at the second and subsequent heatwaves (Table 3). The average daily maximum and minimum temperature during heatwave episodes lasted for 3.2 days had exceeded 86 °F and 68 °F, respectively. For the first heatwave of the season the average number of hospitalization were 4.59 per day for the total of 27 days. For the subsequent heatwaves the mean number of hospitalizations per day were 0.99 per day for the total of 84 days.

The effects of heatwaves on HH were estimated using the results of Models 3 and 4, presented in Table 4. After adjusting for seasonality and weekday effects, the relative risk of HH associated with a heatwave episode was 6.89 [95%CI: 4.84–9.8] (Model 3). The relative risk of HH associated with the first heatwave in a summer season was the highest for first episode: 13.33 [95%CI: 7.4–24] (Model 4). The risk declined to 3.74 [95%CI: 2.43–5.76] for the subsequent heatwave episodes. For the four additional definitions, the relative risks associated with the first heatwave and the subsequent heatwaves are presented in Table 4. The first heatwave has consistently higher relative risks than subsequent heatwaves for all alternative definitions. The measures for the quality of fit for different model specifications and heatwave definitions are presented in Table 5.

## Discussion

The impact of heat on human health has received significant public attention. The most recent *National Climate Assessment* (United States National Climate Assessment (USNCA) Program 2014) and WHO report on heatwaves and health^3^ emphasizes the need for broad public health actions, especially in the areas of preparedness and prevention, which can do much to protect vulnerable population from the detrimental impacts of extreme weather^45^. The heat related mortality has been extensively studied in relation to various death related causes and adaptation scenarios in the context of the current climate and projected climate changes^46^,^47^,^48^,^49^,^50^. The heat related morbidity, on the other hand, has been less examined yet the cost associated with hospitalizations is quite substantial. The analysis demonstrates that a heatwave episode results in almost 7-fold increase in heat-related hospitalizations over 16-year period among the older adults in Boston MSA, a composite of urban and semi-urban communities with mild temperate climate, relatively high living standards, and easy access to medical care.

In this study we utilized medical claims, maintained by the US Centers of Medicare and Medicaid Services. The significant potential of this data repository for conducting a broad range of investigations in environmental epidemiology at the local and nationwide has been widely demonstrated. In our research, we explored CMS data to describe the effects of drinking water contamination on vulnerable population^51^, examined the emerging trends^52^,^53^, seasonal patterns^54^,^55^,^56^, and nationwide spatio-temporal synchronization in hospitalizations due to infectious agents^56^. This data source also allowed us to estimate immediate direct medical expenses associated with hospitalizations directly related to environmental heat exposure. Total charges associated with 41,927 cases of heat-related hospitalizations, reported over 16 years resulted in $438,845,346 nationwide, or ~$27 million annually. In Boston MSA, HHs contributed $5,714,391 of medical charges, which is almost equivalent to the annual state budget allocated to the Supportive Senior Housing of $5.5 million for 2015 (Massachusetts Budget and Policy Center (MassBudget) 2013). The overall impact of heat waves on health is not limited to the heat related morbidity^14^,^57^,^58^. Thus, the observed increase of heat related hospitalizations based on the selected ICD codes is very likely to underestimate the impact of heat waves on health and provides very conservative estimate of the effect and associated costs. As we limited the study to only hospitalizations directly related to environmental heat exposure with well-defined symptoms codes as primary causes, the presented results are likely to be least affected by changes in somewhat complex hospitalization coding rules^58^.

The pattern of hospitalization due to heat varied quite significantly across geographic regions^59^,^60^,^61^,^62^,^63^ and the reasons for such difference are not yet clear. This study demonstrates that one of the reasons might be a different response to heatwaves and its dependence on the timing of heatwaves. While the days defined as heatwave represent only 2% out of 5,844 days under observations, one third of heat-related hospitalizations occurred during one week of the summer-time period annually. The relative risk of heat strokes associated with the first heatwave of a season is 5-fold higher than the risks of the subsequent waves. With the average maximum daily temperature of 87.3 °F and average minimum night temperature of 69.3 °F observed during heatwaves, a sudden increase in daily hospitalization up to 10–15 cases are plausible^14^,^57^,^58^. These findings suggest that the prevention programs should focus their effort on the first heat wave of the season to maximize the public health impact.

**Figure 5:**
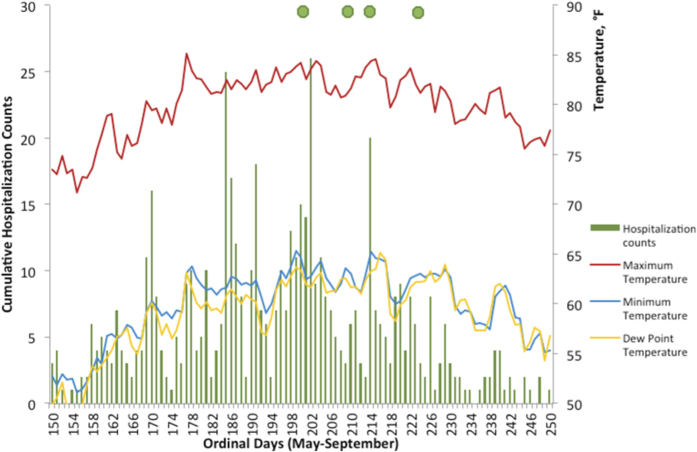
Average daily minimum, maximum, and dew point temperature values (solid lines), average timing of first, second, third and forth heatwave episodes (shown with green dots), and cumulative heat-related hospitalization counts (needle plot) during summer months in Boston MSA, in 1991–2006 (May through September).

We also argue that in the temperate climate of Boston special attention should be paid to daily minimum temperature in setting up public health communication. Our data-derived empirical definition of a heatwave episode includes both minimum and maximum temperature. A day is defined as heatwave if the night-time temperatures is above 65.5 °F (or above 86.5^th^ percentile) for 3 consecutive nights. At this threshold the maximum daily temperature is likely to be about 87.3 °F, or at its 86.5^th^ percentile. Minimum daily air temperature is often used as a proxy variable to estimate average daily near-surface humidity, especially in non-arid climates^64^. The relative near-surface humidity in temperate climate with relative humidity above 50% can be approximated by the conversion formula


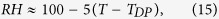


where *RH* is relative humidity, *T* is an ambient temperature, and *T*_*DP*_ is a dew point temperature^65^. From the Eq. (15) follows that the higher dew point temperature indicate higher relative humidity. In Boston MSA, the night-time temperature follows very closely the dew point temperature during summer months (correlation coefficient of 0.86, p < 0.001). The average timing of heatwave episodes tends to cluster when dew points are high late July and August (Fig. 5). Therefore, our empirical definition more likely selects days with high humidity and temperature above 85^th^ percentile, emphasizing that humid nights with high minimum daily temperature are likely to provide little relieve from daily heat.

This study offers a number of methodological innovations for investigating the effects of thermal extremes on human health. In order to define a locally-specific definition of a heatwave episode, we designed an approach that allows incorporating the steep exponential increase in health outcome as daily temperature within a widely accepted linear regression framework and simultaneously select the thresholds for daytime and nighttime temperatures accounting for lag-distributed effects. The use of a threshold assumes that there is a comfort zone, exceeding which human thermoregulation fails to respond properly and adverse health outcomes might occur. We argue that an “ideal” threshold should be both person- and location- specific. A person with underlying health conditions, known to contribute to individual vulnerability^16^,^23^,^66^ might have a temperature threshold lower than that of a healthy individual. Temperature thresholds should be also location-specific and may depend on cultural, social and economic adaptation^67^,^68^. A better understanding of individual-based and community-based thresholds will help to reliably predict an ability to withstand the extreme weather effects and to implement location-specific early warning systems. Furthermore, affordable measures for regular hydration and cooling can be and should be introduced in a timely manner.

The proposed concept of the lag-distributed effect of exposure, initially introduced by Naumova and MacNeill in ref. 55 has been further developed and adapted in this research. The proposed approach accounts for overall duration of exposure by estimating an effective duration using distributed lag model for both minimum and maximum temperature in one model. Diurnal variations between daytime and nighttime convey valuable information on the likelihood of adverse health effects by indicating the potential for heat relief at night after exposure to heat through the day. The smaller difference between minimum and maximum temperatures demonstrates a longer duration of heat exposure and higher relative humidity. This approach allows us to improve the estimation of relative risk by proper depiction of a complex non-linear nature of relationship between temperature and health outcomes and reducing underestimation. In the proposed model each component controls for a biological mechanism or behavioral pattern. The year-related component explicitly controls the temporal changes during multi-year study, including demographic changes and potential adaptation measures due to changes in the coding rules for the medical diagnoses in Medicare claim data^58^. The seasonality-related terms account for sharp intra-year changes in hospitalizations due to environmental exposure to heat, markedly different during warm and cold seasons. The short-term intra-week periodic changes were controlled to recognize the fact that hospital admissions might be influenced by social calendars, so admissions during weekends and holidays are generally lower than during the workdays in the middle of the week. Finally, the two terms of primary interest measure the effect of the first seasonal heatwave and subsequent heatwaves and test the hypotheses that the impact of the first seasonal heat wave differs from the consecutive heatwaves.

As we compared the proposed region-specific data-driven definition of heatwave with four other heatwave definitions, we observed the expected overlap of 69 out of 111 days, with many remaining non-overlapping days differ by a day or two which indicates a good agreement with existing definition as well as certain amount of a site specific localization. The proposed data-driven definition is an improvement over existing schemes as it allows dynamic definition adjusted for local climatic variations and levels of social adaptation. It replaces universal rigid rules with tailored region-specific guidelines that can be adjusted based on underlying latent regional properties, including climate change. This flexibility is important in the context of regional adaptation. The next step is to explore how data-driven location-specific definitions vary across climatic zones and to determine a set of rules for selecting definitions with high predictive ability for specific health conditions and subpopulations.

We strongly advocate for better timing and targeting public health announcements, increasing the awareness of detrimental health effects of heat exposure in older adults and for the need of systematic improvement of living conditions, infrastructure and medical support for most vulnerable population to ensure their wellbeing and reduce the cost of health care. The disproportionally strong impact of the first heatwave highlights the importance of surveillance and early warning systems. The systematic seasonal pattern in heat-related hospitalizations calls for better planning hospital workload in the summer months. It also supports the assertion of the high return of investing in improving early warning notifications of vulnerable population and the urban infrastructure. Computationally intensive mathematical and statistical modeling applied to routinely and timely collected national data should provide strong basis for reliable near-term forecasting and real-time assessment of effectiveness of intervention strategies.

## Additional Information

**How to cite this article**: Liss, A. *et al*. Heat-Related Hospitalizations in Older Adults: An Amplified Effect of the First Seasonal Heatwave. *Sci. Rep.* **7**, 39581; doi: 10.1038/srep39581 (2017).

**Publisher's note:** Springer Nature remains neutral with regard to jurisdictional claims in published maps and institutional affiliations.

## Figures and Tables

**Table 1:** Distribution of cases based on diagnostic code ICD-9-CM.

ICD-9-CM code	Description	Cases (%)
992.0	Heat stroke and sunstroke	115 (16.4)
992.1	Heat syncope	49 (7.0)
992.2	Heat cramps	6 (0.9)
992.3	Heat exhaustion, anhydrotic	40 (5.7)
992.4	Heat exhaustion due to salt depletion	9 (1.3)
992.5	Heat exhaustion, unspecified	465 (66.3)
992.6	Heat fatigue, transient	2 (0.3)
992.7	Heat edema	0 (0)
992.8	Other specified heat effects	7 (1.0)
992.9	Unspecified effects of heat and light	8 (1.1)

**Table 2:** Heatwaves for Boston MSA, 1991–2006: total number of HH cases and days associated with heatwave definitions with Julian calendar days for the first and last heatwaves.

	All*	1991	1992	1993	1994	1995	1996	1997	1998	1999	2000	2001	2002	2003	2004	2005	2006
Proposed Definition																	
Number of HW	44	3	1	2	3	5	4	0	3	4	0	2	2	2	3	4	6
Maximum length	4.64	6	2	4	17	6	2		1	2		3	5	9	1	4	3
Total days	111	11	2	5	22	13	5		3	5		4	7	10	3	10	11
Date of the first HW	195	198	241	188	181	195	165		197	186		215	183	204	216	177	178
Date of the last HW	231	241	242	214	214	228	237		236	251		221	229	224	244	242	213
HH counts**	207	46	0	35	18	17	0		4	7		11	39	3	1	8	18
HH per HW day	1.96	4.18	0	7	0.82	1.31	0		1.33	1.4		2.75	5.57	0.3	0.33	0.8	1.64
HH per non-HW day	0.18	0.16	0.14	0.23	0.25	0.31	0.07	0.17	0.11	0.29	0.09	0.22	0.23	0.05	0.08	0.21	0.25
Definition A																	
Number of HW	81	7	1	5	4	6	2	7	3	10	2	8	7	4	2	9	4
Maximum length	3.75	5	2	5	4	4	1	2	3	5	2	4	9	4	2	6	2
Total days	165	12	2	11	8	11	2	8	6	21	3	15	25	11	3	20	7
Date of the first HW	162	179	143	177	169	170	142	162	196	152	129	123	178	177	161	163	170
Date of the last HW	226	240	144	240	207	229	200	229	237	247	222	222	253	234	243	256	215
HH counts	314	43	2	39	23	30	1	4	10	31	2	27	58	5	2	14	23
HH per HW day	1.74	3.58	1	3.55	2.88	2.73	0.5	0.5	1.67	1.48	0.67	1.8	2.32	0.45	0.67	0.7	3.29
HH per non-HW day	0.14	0.19	0.13	0.21	0.19	0.21	0.06	0.15	0.08	0.14	0.08	0.12	0.11	0.04	0.07	0.18	0.21
Definition B																	
Number of HW	34	2	1	2	2	2	0	3	0	4	0	4	6	2	0	4	2
Maximum length	2.75	4	1	3	2	1		1		3		3	8	2		3	2
Total days	66	5	1	6	4	2		3		7		6	18	4		7	3
Date of the first HW	172	179	144	189	169	196		173		159		123	178	177		177	199
Date of the last HW	209	202	144	240	203	213		199		200		222	253	188		226	215
HH counts	211	35	1	28	20	6		2		15		20	53	5		8	18
HH per HW day	3.18	7	1	4.67	5	3		0.67		2.14		3.33	2.94	1.25		1.14	6
HH per non-HW day	0.17	0.23	0.13	0.28	0.2	0.36	0.07	0.16	0.14	0.24	0.09	0.16	0.14	0.03	0.09	0.2	0.24
Definition C																	
Number of HW	46	1	2	3	3	2	1	2	3	5	1	5	4	3	3	4	4
Maximum length	7.69	6	2	8	14	10	1	3	7	20	4	6	14	11	2	9	6
Total days	237	6	3	15	30	16	1	6	15	31	4	17	30	25	6	23	9
Date of the first HW	186	201	195	190	169	205	223	181	199	154	178	169	177	180	204	163	193
Date of the last HW	226	206	241	244	217	230	223	199	243	252	181	236	235	229	228	226	222
HH counts	290	43	1	39	26	20	0	4	9	17	1	24	62	4	3	15	22
HH per HW day	1.35	7.17	0.33	2.6	0.87	1.25	0	0.67	0.6	0.55	0.25	1.41	2.07	0.16	0.5	0.65	2.44
HH per non-HW day	0.15	0.18	0.14	0.22	0.2	0.3	0.07	0.15	0.09	0.27	0.09	0.14	0.08	0.05	0.07	0.17	0.22
Definition D																	
Number of HW	9	1	0	1	1	0	0	0	0	2	0	1	2	0	0	0	1
Maximum length	1.71	1		2	1					1		2	4				1
Total days	14	1		2	1					2		2	5				1
Date of the first HW	200	202		190	203					187		221	185				215
Date of the last HW	209	202		191	203					200		222	230				215
HH counts	97	18		24	2					9		9	30				5
HH per HW day	7.43	18		12	2					4.5		4.5	6				5
HH per non-HW day	0.22	0.34	0.14	0.3	0.32	0.4	0.07	0.17	0.14	0.27	0.09	0.23	0.29	0.07	0.09	0.25	0.32

^a^The maximum length, date of the first and last heatwave, heat-related hospitalizations (HH) per heatwave (HW) and non-HW days across all years are shown as averages; Number of HW episodes, total number of days associated with HW and total HH counts across all years are shown as sum of all events.

^b^HH counts are estimated over the summer period of 151 days from May to September.

**Table 3:** Descriptive statistics of heatwave episodes occurrence for Boston MSA, 1991–2006: total duration and average timing; heat-related hospitalizations (HH); average daytime (Max T) and night time (Min T) temperature values during the heatwave episodes and relative risks with CI_95%_ of HH associated with heatwave episodes estimated from Model 3.

Heatwave sequence	Number seasons	Number of days	Mean (SD) Ordinal Day	Number of HH	HH per day	Max Temp °F Mean (SD)	Min Temp °F Mean (SD)	HW episode RR (CI_95%_)
1	1	27	196.7 (17.6)	124	4.59	87.4 (6.1)	68.8 (2.5)	15.3 (9.2–25.5)
2	4	39	213.7 (14.0)	42	1.08	87.3 (4.7)	69.1 (2.1)	3.8 (2.1–6.7)
3	4	33	214.3 (13.0)	30	0.91	87.2 (4.6)	68.9 (2.1)	2.7 (1.5–5)
4+	5	12	226.6 (17.2)	11	0.92	84.0 (5.3)	68.6 (2.3)	7.1 (2.7–18.9)

**Table 4:** The results of the sequentially built Negative-Binomial Generalized Linear Models (NB-GLM) with harmonic terms for heat-related hospitalizations (HH) for heatwave episodes in Boston MSA, 1991–2006 based on the proposed definition and four alternative definitions of a heatwave episode.

	Model 2			Model 3			Model 4		
	Estimate	Std. Error	p-value	Estimate	Std. Error	p-value	Estimate	Std. Error	p-value
Proposed Definition									
Intercept^a^	−2.46	0.20	<0.001	−2.76	0.20	<0.001	−2.88	0.20	<0.001
COS (1^st^)^b^	−1.58	0.11	<0.001	−1.38	0.10	<0.001	−1.38	0.10	<0.001
** **SIN (1^st^)	0.07	0.12	0.52	0.14	0.11	0.21	0.13	0.11	0.25
** **COS (2^nd^)	0.69	0.09	<0.001	0.59	0.09	<0.001	0.56	0.09	<0.001
** **SIN (2^nd^)	0.46	0.10	<0.001	0.21	0.10	0.03	0.23	0.09	0.02
** **WEEKEND-HDAY^c^	−0.23	0.12	0.06	−0.26	0.12	0.03	−0.29	0.12	0.01
** **HW Episode				1.93	0.18	<0.001			
** **1st HW							2.59	0.30	<0.001
** **Later HW							1.32	0.22	<0.001
Definition A									
** **HW Episode				2.26	0.14	<0.001			
** **1st HW							2.48	0.28	<0.001
** **Later HW							2.20	0.15	<0.001
Definition B									
** **HW Episode				2.52	0.19	<0.001			
** **1st HW							2.63	0.34	<0.001
** **Later HW							2.46	0.22	<0.001
Definition C									
** **HW Episode				1.70	0.15	<0.001			
** **1st HW							2.20	0.20	<0.001
** **Later HW							1.21	0.19	<0.001
Definition D									
** **HW Episode				2.84	0.44	<0.001			
** **1st HW							3.01	0.53	<0.001
** **Later HW							2.21	0.77	<0.001

^a^All models are adjusted for the year effects as described in Method section (data not shown).

^b^The *sin* and *cos* terms for the first and second harmonics of seasonal components, respectively.

^c^A term for weekend and holiday effects (WEEKEND-HDAY).

**Table 5:** Quality of fit measures for different model specifications and heatwave definitions.

Model	AIC*	Null Deviance (DF = 5833)	Residual Deviance (DF)	−(Log-Likelihood)
Model 2	3343	2659.3	1580.6 (5813)	3299
Model 3	3221	2772.9	1622.1 (5812)	3175
Model 4				
Proposed Definition	3204	2772.9	1616.3 (5809)	3152
Definition A	3100	2956.2	1620.0 (5811)	3052
Definition B	3167	2838.5	1625.1 (5811)	3119
Definition C	3211	2668.1	1573.4 (5811)	3163
Definition D	3275	2532.9	1557.2 (5811)	3226

^*^AIC–Akaike Information Criterion; DF–degrees of freedom.
